# Determinants of infant mortality in the Jequitinhonha Valley and in the North and Northeast regions of Brazil

**DOI:** 10.1590/S1518-8787.2017051006391

**Published:** 2017-02-23

**Authors:** Maria do Carmo Leal, Sonia Duarte de Azevedo Bittencourt, Raquel Maria Cardoso Torres, Roberta Pereira Niquini, Paulo Roberto Borges de Souza

**Affiliations:** I Departamento de Epidemiologia e Métodos Quantitativos em Saúde. Escola Nacional de Saúde Pública Sergio Arouca. Fundação Oswaldo Cruz. Rio de Janeiro, RJ, Brasil; II Programa de Pós-Graduação em Saúde Pública. Escola Nacional de Saúde Pública Sergio Arouca. Fundação Oswaldo Cruz. Rio de Janeiro, RJ, Brasil; III Instituto Federal de Educação, Ciência e Tecnologia do Rio de Janeiro. Rio de Janeiro, RJ, Brasil; IV Laboratório de Informações em Saúde. Instituto de Comunicação e Informação Científica e Tecnológica em Saúde. Fundação Oswaldo Cruz. Rio de Janeiro, RJ, Brasil

**Keywords:** Infant Death, Risk Factors, Socioeconomic Factors, Prenatal Care, Case-Control Studies, Infant Mortality, Health Inequalities

## Abstract

**OBJECTIVE:**

This study aims to identify the social and demographic determinants, in addition to the determinants of reproductive health and use of health services, associated with infant mortality in small and medium-sized cities of the North, Northeast and Southeast regions of Brazil.

**METHODS:**

This is a case-control study with 803 cases of death of children under one year and 1,969 live births (controls), whose mothers lived in the selected cities in 2008. The lists of the names of cases and controls were extracted from the *Sistema de Informação sobre Mortalidade* (SIM – Mortality Information System) and the *Sistema de Informação sobre Nascidos Vivos* (SINASC – Live Birth Information System) and supplemented by data obtained by the research of “active search of death and birth”. Data was collected in the household using a semi-structured questionnaire, and the analysis was carried out using multiple logistic regression.

**RESULTS:**

The final model indicates that the following items are positively and significantly associated with infant mortality: family working in agriculture, mother having a history of fetal and infant losses, no prenatal or inadequate prenatal, and not being associated to the maternity hospital during the prenatal period. We have observed significant interactions to explain the occurrence of infant mortality between race and socioeconomic score and between high-risk pregnancy and pilgrimage for childbirth.

**CONCLUSIONS:**

The excessive number of home deliveries and pilgrimage for childbirth indicates flaws in the line of maternity care and a lack of collaboration between the levels of outpatient and hospital care. The study reinforces the need for an integrated management of the health care networks, leveraging the capabilities of cities in meeting the needs of pregnancy, delivery and birth with quality.

## RESUMO

**OBJETIVO:**

Identificar os determinantes sociais, demográficos, da saúde reprodutiva e de utilização dos serviços de saúde associados ao óbito infantil em municípios de pequeno e médio porte das regiões Norte, Nordeste e Sudeste do Brasil.

**MÉTODOS:**

Trata-se de um estudo caso-controle com 803 casos de óbito de menores de um ano e 1.969 nascidos vivos (controles), cujas mães residiam em 2008 nos municípios selecionados. As listas nominais dos casos e do controles foram extraídas do Sistema de Informação sobre Mortalidade e do Sistema de Informação sobre Nascidos Vivos e completadas por dados obtidos pela pesquisa de “busca ativa de óbito e nascimento”. A coleta de dados foi realizada em domicílio por meio de questionário semiestruturado, e a análise, por meio de regressão logística múltipla.

**RESULTADOS:**

O modelo final indicou que estão associadas positivamente e significativamente ao óbito infantil: a família trabalhar na agricultura, a mãe ter tido história de perdas fetais e infantis, não ter feito pré-natal ou ter tido um pré-natal inadequado e não estar vinculada à maternidade durante o pré-natal. Foram observadas interações significativas para explicar a ocorrência do óbito infantil entre cor de pele e escore socioeconômico e entre gestação classificada como de risco e peregrinação para o parto.

**CONCLUSÕES:**

O número excessivo de partos domiciliares e de peregrinação para o parto indica falhas na linha de cuidado da gestante e desarticulação entre os níveis de atenção ambulatorial e hospitalar. O estudo reforça a necessidade de uma gestão integrada das redes de atenção à saúde, potencializando as capacidades municipais em atender, com qualidade, à gestação, ao parto e ao nascimento.

## INTRODUCTION

Although the coefficient of infant mortality in Brazil shows a relevant decrease, there are still obstacles to be overcome, such as the inequalities related to socioeconomic development and access to health services. In addition, the country also face problems related to the coverage of vital events, a fact that affects the knowledge of the extent of infant mortality, as well as the identification of associated factors.

The risks of infant mortality are higher in some areas of the Southeast region, such as in the Jequitinhonha Valley, and the North and Northeast regions, especially in small and medium-sized cities. These cities have a high concentration of poverty, more barriers to the access to health services^4,15,a^ and problems of completeness of vital data in the *Sistema de Informação sobre Mortalidade* (SIM – Mortality Information System) and the *Sistema de Informação sobre Nascidos Vivos* (SINASC – Live Birth Information System).

In this sense, the knowledge on the set of factors of the complex network that determines infant mortality can positively guide the efforts specifically focused on the reduction of infant mortality in regions with worse social and health indicators and with major problems related to the quality of the vital data.

This study has aimed to identify the social and demographic determinants, in addition to the determinants of reproductive health and use of health services, associated with infant mortality.

## METHODS

This is a case-control study conducted with a sub-sample of cities of the research “Estimação da cobertura dos sistemas de informações sobre nascidos vivos (SINASC) e sobre mortalidade (SIM) e da mortalidade infantil por Unidade da Federação, 2008”, henceforth referred to as “active search of death and birth”^b^. This sub-sample included seventy-five cities of up to 200,000 inhabitants, located in the North, Northeast and Southeast regions (specifically the Jequitinhonha Valley in the Southeast region, in the State of Minas Gerais), Brazil.

The selected cities were classified into three strata of population category (up to 20,000; from 20,001 to 50,000; from 50,001 to 200,000 inhabitants) and of adequacy of vital information, according to the method developed by Andrade and Szwarcwald^4^. We selected all eligible cities (n = 36) from the first population stratum, while we chose twenty and nineteen cities from the second and third strata, respectively, with probability of selection proportional to population size. We calculated the sample expansion factors by the inverse of the probability of selection of each city in each stratum, and then we adjusted the weights, i.e., calibrated them, so that the estimates in each stratum were consistent with the total number of live births (corrected by the process of active search).

The data of the 2010 Census^b^ show that, among the seventy-five cities studied, the proportion of urban population ranged from 20.3% (in a city in the State of Pará) to 99.3% (in a city in the metropolitan region of Fortaleza, State of Ceará). In almost a quarter of the cities visited, the urban population represented less than 50% of the total population. The average gross domestic product (GDP) per capita of the studied cities was three and a half times lower than the GDP of Brazil, although, among the cities, there are some petrochemical poles and large mining companies.

In 84% of the cities, more than 50% of the resident population had *per capita* household income of up to half a minimum wage. Almost half of the cities have low human development index (HDI). The median coverage of the sewage system was only 17.4%, well below the water supply (66.7%)^c^.

The lists of the names of the children who died under one year and the live births of mothers living in the cities selected, which was provided by the Secretariat of Health Surveillance, Ministry of Health (SVS-MS), for the period from January 1 to December 31, 2008, were supplemented by data collected in the research of “active search of death and birth”.

We defined as cases all children who died under one year present in the updated list of deaths. For each death, we selected, using a random drawing, two controls present in the updated list of live births. The controls that were not located were replaced by controls living in the neighborhood.

To obtain the data, we carried out household interviews with the mother or responsible for the newborn, between April and December, 2010. The questions of the semi-structured questionnaire, specifically related to 2008, focus on: socioeconomic conditions of the family, demographic characteristics of the mothers, health care of the mother (prenatal - PN, childbirth, puerperium), and health conditions of the newborn (after the delivery until the first year of life). The data collection tool was tested in a pilot study and the field staff responsible for applying it was composed of nine supervisors and twenty-five interviewers, all properly trained.

The independent variables selected for the study include: demographic characteristics of the mother [age (10 to 19, 20 to 34, and ≥ 35 years), race (white, black, brown, yellow or native), education of the mother (none or incomplete elementary school, complete elementary school or incomplete high school, complete high school, or complete or incomplete higher education), marital status (no partner, with partner but he does lives together, and lives with partner)], behaviors during pregnancy (smoking habit and alcohol consumption), and socioeconomic conditions of the family (housing adequacy according to the classification adopted by the Brazilian Institute of Geography and Statistics – IBGE: adequate – households that have water supply, sewage or rainwater network or septic tank, and direct or indirect garbage collection; inadequate – other households^d^; socioeconomic score of the *Associação Brasileira de Empresas de Pesquisa* (ABEP - Brazilian Association of Research Companies)^5^ – Classes A/B/C and D/E; and working in agriculture – yes, no).

The variables of reproductive health were: obstetric history (abortion history, history of fetal or infant losses, previous deliveries with loss, and first time pregnancy) and hospitalization during pregnancy (yes, no). Pregnant women were classified as of high-risk (yes, no) according to the self-reported presence of at least one of the following conditions: hypertension, heart disease, diabetes, syphilis, human immunodeficiency virus (HIV), Rh incompatibility, threatened abortion, seizures, hospitalization because of obstetric causes, or no prenatal care.

The adequacy of prenatal care was created by the grouping of the categories of the Kotelchuck index^7^: (i) no PN or inadequate PN, and (ii) adequate PN or more than adequate. This score was adapted according to the recommendation of the Ministry of Health, in which, a PN considered as adequate must start before the 16th gestational week, and at least one appointment must be performed in the first quarter, two in the second quarter and three in the third quarter^9,e^. The remaining women were classified as inadequate PN or no PN.

We also considered the following independent variables: association of the pregnant mother to the maternity hospital during the prenatal period (yes, no), need to search for more than one service unit at the time of hospitalization for delivery, named “pilgrimage” (yes, no, delivery at home), and who performed the delivery (delivery by the mother herself, midwife, healthcare professional, and other).

We measured the intensity of the associations between each independent variable and the dependent variable by simple logistic regression, calculating the estimates of odds ratio (OR) and 95% confidence intervals (95%CI). We tested the presence of interaction between high-risk pregnancy and pilgrimage for childbirth, and between race and socioeconomic score.

In the following step, we performed multiple logistic regression models using the manual stepwise method, testing the inclusion and exclusion of variables and significant interactions at the 20% level in the simple logistic regression analysis. We kept all variables and significant interactions at the 5% level in the final multiple model. After the choice of the final model, we calculated the adjusted estimates of OR and the respective 95%CI. We excluded yellow and native women from this stage of analysis, given the extremely low sample size for adequate multiple modeling.

All statistical analyses were carried out using the software R 2.15.3.

This study was approved by the Research Ethics Committee of the Centro de Pesquisa René Rachou (Protocol 206/2009). All participants signed the informed consent.

## RESULTS

Of the seventy-five cities of the sample, we excluded seven (9%) cities from the analysis because the health departments confirmed no infant death in 2008.

Of the 942 infant deaths occurred in the sixty-eight cities studied, 818 (86.8%) cases were included in this study. The losses were due to the address not being located, the family moving to another city, and the family refusing to participate in the research. After calibration, the estimated number of deaths was 803 and the number of controls was 1,969.

Most of the deaths (66.9%) occurred in the neonatal period, being 53.1% in the first six days of the child’s life and 13.8% between seven and twenty-seven days of life. Of the total cases, 49.3% were preterm and 46.8% had low birth weight. Among the controls, that proportion was 7% and 7.5%, respectively.

Table 1 presents the independent demographic and socioeconomic variables in the simple logistic regression analysis to explain infant mortality. The following variables showed positive association with infant mortality: education being less than complete high school, smoking habit, belonging to families who worked in agriculture, and being from the economic classes D or E.

**Table 1:** Demographic and socioeconomic characteristics of the mothers of the cases and controls in a sample of small and medium-sized cities in the North and Northeast regions of Brazil and in the Jequitinhonha Valley, State of Minas Gerais, Brazil, 2011.

Variable	Case			Control			OR	95%CI
	
	n	%	95%CI	n	%	95%CI		
Age of the mother								
10 to 19 years	93	12.1	9.2–15.9	209	10.7	8.6–13.3	1.12	0.77–1.63
20 to 34 years	586	75.9	72.1–79.3	1,467	75.3	71.7–78.6	1.00	
35 years or more	93	12.0	8.9–16.0	272	13.9	11.1–17.3	0.86	0.58–1.27
Race								
White	145	18.1	15.0–21.7	292	14.9	12.6– 17.4	1.00	-
Black	47	5.9	3.7–9.2	125	6.4	4.8–8.5	0.75	0.46–1.24
Brown	593	74.1	70.3–77.6	1,503	76.5	73.2–79.5	0.80^a^	0.63–1.01
Yellow or native	15	1.9	0.9–4.1	45	2.3	1.4– 3.7	0.70	0.26–1.85
Education of the mother								
None or incomplete elementary school	424	54.2	48.4–60.0	825	42.0	38.3–45.8	1.86^a^	1.39–2.48
Complete elementary school or incomplete high school	123	15.7	12.4–19.7	289	14.7	12.5–17.3	1.54^a^	1.06–2.22
Complete high school or complete or incomplete higher education	235	30.1	25.5–35.0	849	43.3	39.6–47.0	1.00	-
Marital Status								
No partner	137	17.1	13.3–21.8	337	17.1	14.5–20.1	0.98	0.73–1.32
With partner. but he does not live together	46	5.8	3.7–8.9	141	7.1	5.5–9.2	0.79	0.46–1.36
Lives with partner	617	77.1	72.8–80.9	1,490	75.7	72.4–78.8	1.00	-
Smoking habit	116	14.5	11.1–18.8	176	9.0	6.7–11.8	1.73^a^	1.18–2.54
Drinking habit	186	23.4	18.1–29.6	350	17.8	14.7–21.4	1.41^a^	0.94–2.12
Works in agriculture	273	34.0	27.9–40.6	486	24.7	19.9–30.2	1.57^a^	1.20–2.05
ABEP score								
Class D+E	568	70.7	65.6–75.4	1,064	54.1	49.3–58.7	2.05^a^	1.59–2.64
Class A+B+C	235	29.3	24.6–34.4	905	45.9	41.3–50.7	1.00	-
Housing conditions								
Inadequate	614	77.0	71.1–82.0	1,513	76.9	71.3–81.6	1.01	0.77–1.32
Adequate	183	23.0	18.0–28.9	455	23.1	18.4–28.7	1.0	-

ABEP: *Associação Brasileira de Empresas de Pesquisa* (Brazilian Association of Research Companies).

^a^ p ≤ 0.20.

Infant mortality was also positively associated with: obstetric history of fetal or infant losses, no PN or inadequate PN, no association to the maternity hospital during the prenatal period, high-risk pregnancy, hospitalization during pregnancy, pilgrimage or delivery at home, and delivery by the mother herself (Table 2).

**Table 2:** Reproductive history and prenatal and delivery care of the cases and controls in a sample of small and medium-sized cities in the North and Northeast regions of Brazil and in the Jequitinhonha Valley, State of Minas Gerais, Brazil, 2011.

	Case			Control			OR	95%CI
	
	n	%	95%CI	n	%	95%CI		
Obstetric history								
First time pregnancy	282	35.3	30.8–40.2	737	37.5	34.3–40.8	0.92	0.72–1.17
Abortion history	158	19.8	15.2–25.3	425	21.6	19.3–24.1	0.89	0.62–1.29
History of fetal and infant losses	56	7.0	5.2–9.2	76	3.9	2.8–5.3	1.75^c^	1.20–2.56
Previous deliveries without loss	303	37.9	33.3–42.8	731	37.0	34.2–40.0	1.00	-
Adequacy of prenatal carea								
Inadequate or no prenatal care	297	40.2	35.2–45.4	497	26.1	22.9–29.6	1.90^c^	1.54–2.34
Adequate or more than adequate	442	59.8	54.6–64.8	1,405	73.9	70.4–77.1	1.00	
Association to the maternity hospital during the prenatal period								
No	431	60.3	55.1–65.3	1,047	54.3	50.5–58.0	1.28^c^	1.02–1.61
Yes	283	39.7	34.7–44.9	882	45.7	42.0–49.5	1.00	
High-risk pregnancy^b^	409	50.9	43.4–58.4	531	27.0	23.4–30.9	2.80^c^	1.98–3.96
Admission during pregnancy	159	19.8	15.3–25.3	270	13.7	11.2–16.6	1.56^c^	1.06–2.28
Pilgrimage for childbirth								
Delivery at home	63	7.9	4.7–13.0	48	2.5	1.6–3.7	3.72^c^	2.12–6.52
Pilgrimaged	132	16.4	12.0–22.0	190	9.7	7.5–12.4	1.97^c^	1.39–2.79
Did not pilgrimage	607	75.7	70.8–80.1	1,730	87.9	85.1–90.2	1.00	-
Who performed the delivery								
Health professional	681	87.7	82.9–91.3	1,802	92.3	88.7–94.9	1.00	-
Midwife	42	5.4	3.2–8.8	104	5.3	3.0–9.4	1.06	0.60–1.86
Delivery by the mother	40	5.2	2.9–9.0	28	1.4	0.8–2.6	3.79^c^	1.61–8.91
Other	14	1.8	0.7–4.5	18	0.9	0.4–2.0	2.02^c^	0.69–5.88

^a^ Indicator proposed by Leal et al.^9^

^b^ According to the criteria defined in the study and described in the Methods section.

^c^ p ≤ 0.20.

The multiple logistic regression indicated that the following items are positively associated with infant mortality: family working in agriculture, mother having a history of fetal and infant losses before the pregnancy under study, no PN or an inadequate PN, and not being associated to the maternity hospital during the prenatal period. The smoking habit presented a borderline significance, but we kept it in the model because of its epidemiological relevance (Table 3).

**Table 3:** Adjusted analysis of the factors associated with infant mortality in a sample of small and medium-sized cities in the North and Northeast regions of Brazil and in the Jequitinhonha Valley, State of Minas Gerais, Brazil, 2011.

Variable	OR	95%CI
Among white mothers		
ABEP score - Classes D+E	3.05	1.74–5.34
ABEP score - Classes A+B+C	1	
Among black mothers		
ABEP score - Classes D+E	1	0.46–2.17
ABEP score - Classes A+B+C	1	-
Among brown mothers		
ABEP score - Classes D+E	1.55	1.12–2.15
ABEP score - Classes A+B+C	1	-
Smoking habit		
Yes	1.49	0.99–2.23
No	1	
Works in agriculture		
Yes	1.55	1.12–2.15
No	1	
Obstetric history		
First time pregnancy	1.09	0.79–1.50
Abortion history	0.80	0.51–1.28
History of fetal and infant losses	1.77	1.12–2.81
Previous deliveries without loss	1	-
Adequacy of prenatal care^a^		
Inadequate or no prenatal care	1.41	1.07–1.86
Adequate or more than adequate	1	-
Association to the maternity hospital during prenatal		
No	1.42	1.10–1.82
Yes	1	-
High-risk pregnancy^b^		
Delivery at home	7.63	2.36–24.65
Pilgrimaged	2.71	1.57–4.68
Did not pilgrimage	1	-
Pregnancy not at risk^b^		
Delivery at home	1.76	0.68–4.53
Pilgrimaged	1.06	0.54–2.06
Did not pilgrimage	1	-

ABEP: *Associação Brasileira de Empresas de Pesquisa* (Brazilian Association of Research Companies)

^a^ Indicator proposed by Leal et al.^9^

^b^ According to the criteria defined in the study and described in the Methods section.

We observed significant interactions between race and socioeconomic score and between high-risk pregnancy and pilgrimage (Table 3).

Mothers of cases of infant death had higher chance of belonging to the economic classes D or E when compared to the mothers of the controls, considering both white and brown mothers. However, this association was not observed among black women (Table 3).

Only in the group of high-risk pregnancy the cases presented a higher chance of delivery at home and pilgrimage when compared to the controls. This association was not statistically significant in the group that had no risk (Table 3).

## DISCUSSION

Most of the cities included in this study are located in remote areas, being these cities small and medium-sized, with a large population living in rural areas and in poverty^c^. Simultaneously, they have poor welfare indicators and perinatal results, which are almost always invisible because of the incompleteness of the data (among them, the vital data). A highlight of this study is the visibility given to the health conditions of the population neglected in these cities.

The partnership established between this research and the active search of death and live births, conducted preliminary by Szwarcwald et al.^b^, has allowed us to know the actual number of deaths and live births in the cities studied and carry out a case-control study with minimization of selection biases.

Inherent to the nature of case-control studies, we cannot rule out recall bias. Mothers of deceased children may have valued more their health problems in relation to the mothers of controls. Additionally, another information bias may have happened, such as the omission of certain risk behaviors (unhealthy habits or few prenatal appointments, for example) because they somehow feel guilty because of the death^13^.

Regarding the determinants of infant mortality, similar to that observed in other studies carried out in Fortaleza, CE, and Campinas, SP, Brazil, the age of the mother was not a risk factor for infant mortality^2,11^. Almeida et al.^2^ have also observed, in bivariate analysis, an association between the level of education of the mother and infant mortality, which disappeared in the presence of the other variables included in the model. In this study, although the families of the cases and controls share similar life conditions and access to local health services, the mothers of the children who died were even poorer, which corroborates what has been observed in studies carried out in Salvador, BA, Fortaleza, CE, and Campinas, SP^2,6,13^.

We observed a greater chance of infant death among mothers with a history of fetal and infant loss, classified as of obstetric risk and who did not have adequate prenatal, similarly to that described in previous studies^1,2,13^. The presence of maternal morbidities, such as hypertension and diabetes, and negative outcomes in previous pregnancies (fetal losses and infant deaths) have been linked to negative results in several studies^1,13^. These social determinants of family and reproductive history of the mother have already been widely disseminated in the scientific literature^8,15^ and incorporated as gestational risk criteria in the healthcare protocols of the Ministry of Health^f^.

The most astonishing datum is the continued invisibility of the women at high risk during prenatal monitoring, as 74.6% of them went to basic care, without being referred to specialized services. This percentage was similar to that observed in women classified as having habitual obstetric risk (80.2%) (data not shown). These data indicate shortcomings in the care of pregnant women, possibly because of the absence of detection and treatment of adverse pregnancy conditions^12^.

Studies carried out in different geographic areas have shown that women at high risk reported greater difficulty with appointments related to PN reference services^13,14,16^.

Regarding the collaboration between the levels of outpatient and hospital care for delivery care, we could also see that the absence of an association between the pregnant women and the maternity hospital was an important risk factor for infant mortality in these small and medium-sized cities with large rural population. Therefore, we highlight the need to organize the healthcare network, so as to ensure the timely access, with safe movement, to centers of greater complexity to meet this demand^3^.

This lack of organization of the healthcare network must have contributed to the excessive number of deliveries at home, made by the mother herself or by lay midwives. It is important to highlight that the occurrence of a delivery at home is not, in itself, a risk factor for infant mortality. In countries that provide assistance to deliveries at home carried out by professional midwives, the decision on the place of birth is the right of the woman, except for first time pregnancies^2^. However, in the cities studied, we could see almost eight times more deliveries at home in the presence of obstetric risk among the cases of infant death when compared to the controls.

Another result of the lack of organization of the healthcare network is the substantial number of women who needed to search, using their own resources, for more than one maternity hospital for their care. This phenomenon, called pilgrimage for childbirth, occurred almost three times more among the cases of infant death when it involved women with obstetric risk when compared to the controls. As noted by the literature^10^, the pilgrimage becomes even worse when we consider the maternal risk.

It is known that the coverage of prenatal and childbirth care is almost universal in Brazil^g^. However, there are deficiencies in the quality of the process and disparities in the coverage of the care according to socioeconomic class and place of residence^12,16^, which probably contributes to the maintenance of the high rates of infant mortality.

The analyses carried out in small and medium-sized cities show significant difficulties in the access to the healthcare network, expressed in the search for more than one maternity hospital and the excessive number of deliveries at home, without assistance of a qualified professional. As shown in this study, these facts have increased the chance of infant deaths and require the implementation of timely and safe transport systems for pregnant women or the building of homes for pregnant women and recent mothers next to hospitals. Another recommendation is the greater compliance of health professionals to the healthcare protocols of the Ministry of Health, with risk classification and appropriate referral of pregnant women according to their needs^8^.

At a broader level, the results of this research reinforce the need for an integrated management of the healthcare networks, leveraging the capabilities of cities in meeting the needs of pregnancy, delivery and birth with quality.

## Footnotes

a Lansky S, França E, Ishitani L, Perpétuo IHO. Evolução da mortalidade infantil no Brasil – 1980 a 2005. In: Ministério da Saúde (BR), Secretaria de Vigilância em Saúde, Departamento de Análise de Situação em Saúde. Saúde Brasil 2008: 20 anos de Sistema Único de Saúde (SUS) no Brasil. Brasília (DF); 2009. p.239-65.

b Szwarcwald CL, Morais Neto OL, Frias PG, Souza Junior PRB, Escalante JJC, Lima R. B. Busca ativa de óbitos e nascimentos no Nordeste e na Amazônia Legal: Estimação das coberturas do SIM e do Sinasc nos municípios brasileiros. In: Brasil. Ministério da Saúde. Saúde Brasil 2010: uma análise da situação de saúde e de evidências selecionadas de impacto de ações de vigilância em saúde, Brasília, p. 372, 2011.

c Instituto Brasileiro de Geografia e Estatística. Censo Demográfico 2010: características da população e dos domicílios: resultados do universo. Rio de Janeiro; 2011.

d Instituto Brasileiro de Geografia e Estatística. Censo Demográfico 2010: características urbanísticas do entorno dos domicílios. Rio de Janeiro; 2012.

f Ministério da Saúde (BR), Secretaria de Atenção à Saúde, Departamento de Ações Programáticas Estratégicas. Pré-natal e puerpério: atenção qualificada e humanizada: manual técnico. Brasília (DF); 2006. (Série A. Normas e Manuais Técnicos) - (Série Direitos Sexuais e Direitos Reprodutivos, caderno 5).

g Ministério da Saúde (BR); Centro Brasileiro de Análise e Planejamento. Pesquisa Nacional de Demografia e Saúde da Criança e da Mulher – PNDS 2006: dimensões do processo reprodutivo e da saúde da criança. Brasília (DF); 2009. (Série G. Estatística e Informação em Saúde).
